# PROTOCOL FOR LIVER TRANSPLANTATION IN HILAR CHOLANGIOCARCINOMA

**DOI:** 10.1590/0102-672020210002e1618

**Published:** 2021-12-17

**Authors:** Lucas ERNANI, Rodrigo Bronze de MARTINO, Wellington ANDRAUS, Eduardo de Souza Martins FERNANDES, Felipe Pedreira Tavares de MELLO, Ronaldo ANDRADE, Leandro Savattone PIMENTEL, Luciana Bertocco de Paiva HADDAD, Fabricio Ferreira COELHO, Paulo HERMAN, Luiz Augusto Carneiro D’ALBUQUERQUE

**Affiliations:** 1 Department of Gastroenterology, Hospital de Clínicas, Medical School, University of São Paulo, São Paulo, SP, Brazil; 2 Department of General Surgery and Transplantation, Hospital Adventista Silvestre, Rio de Janeiro, RJ, Brazil; 3 Abdominal Transplant Surgery, Hospital São Lucas - Copacabana, Rio de Janeiro, RJ, Brazil

**Keywords:** Transplantation, Liver transplantation, Cholangiocarcinoma, Klatskin tumor, Transplante, Transplante de fígado, Colangiocarcinoma, Tumor de Klatskin

## Abstract

**Background:**

Hilar cholangiocarcinoma represents more than half of all cholangiocarcinoma cases, having poor prognosis and presenting a median overall survival after diagnosis of 12-24 months. In patients who have unresectable tumors with a better prognosis, the proposal to perform liver transplantation emerged for expanding the possibility of free margins by performing total hepatectomy.

**Aim:**

To provide a Brazilian protocol for liver transplantation in patients with hilar cholangiocarcinoma.

**Method:**

The protocol was carried out by two Brazilian institutions which perform a large volume of resections and liver transplantations, based on the study carried out at the Mayo Clinic. The elaboration of the protocol was conducted in four stages.

**Result:**

A protocol proposal for this disease is presented, which needs to be validated for clinical use.

**Conclusion:**

The development of a liver transplantation protocol for cholangiocarcinoma aims not only to standardize the treatment, but also enable a better assessment of the surgical results in the future.

## RESUMO

**Racional:**

O colangiocarcinoma hilar representa mais da metade de todos os casos de colangiocarcinoma; tem prognóstico reservado e sobrevida global mediana de 12- 24 meses após o diagnóstico. A proposta de realizar transplante hepático surgiu para ampliar a possibilidade de margens livres através de hepatectomia total nos portadores de tumoresirressecáveis com melhor prognóstico.

**Objetivo:**

Apresentar protocolo brasileiro para realização de transplante hepático em pacientes com colangiocarcinoma hilar.

**Método:**

O protocolo foi realizado por duas instituições com grande volume de ressecções e transplantes hepáticos no Brasil, baseado no trabalho realizado pela MayoClinic. A elaboração foi dividida em quatro etapas.

**Resultado:**

É apresentada proposta de protocolo para esta doença a ser validada na aplicação clínica.

**Conclusão:**

Foi possível elaborar protocolo de transplante hepático para colangiocarcinoma a fim de uniformizar o tratamento e melhor avaliar os resultados cirúrgicos.

## INTRODUCTION

Cholangiocarcinoma (CCA) is the second most common primary neoplasm of the liver after hepatocellular carcinoma, accounting for 10-15% of all hepatobiliary neoplasms¹. CCA originates from the biliary epithelium, histologically consisting of an adenocarcinoma in 95% of the cases. Its classification, used in clinical practice, is based on the anatomical location of the tumor and is divided into intrahepatic and extrahepatic. The latter includes hilar and distal (inferior lower third of the choledochal) cholangiocarcinomas^6^. 

Hilar cholangiocarcinoma (hCCA) - also known as Klatskin tumor - is the most frequent type of CCA, representing more than half of all CCA cases. It has a poor prognosis, with a median overall survival after diagnosis of 12-24 months^6^. The most important prognostic factor for this tumor is achieving free margins through surgical resection; however, it is only achieved in 25-40% of patients. Moreover, the overall survival in patients who underwent R0 resection is 40-45% in 5 years^6^ ^,^ ^10^ ^,^ ^12^. 

The proposal of performing liver transplantation (LT) in this group of patients emerged in the late 1980s and early 1990s. The justification for this pioneering idea regarded an increase in the number of patients with free margins by performing total hepatectomy in tumors considered unresectable. Initial results were not promising, with a high rate of recurrence (51-53%) and overall survival of 23-30% in 5 years^9^ ^,^ ^14^ ^,^ ^17^. Organ scarcity combined with poor initial results justified the contraindication of hCCA for LT at that time. Based on the data currently available, it is possible to observe that there is benefit in performing LT for hCCA as long as careful candidate selection is carried out^5^ ^,^ ^21^. The current recommendation of the International Liver Transplant Society (ILTS) is to perform LT with an specific protocol for hCCA^18^. 

Therefore, the aim of our study is to present a protocol proposal to guide the clinical use of LT in hCCA. This protocol needs to be validated in future studies. 

## METHODS

This protocol was performed by two high-volume centers of liver transplantation (LT) and liver resection (LR) in Brazil: University Hospital of the Medical School of the University of São Paulo (HCFMUSP) and Hospital Adventista Silvestre/ Hospital São Lucas. The elaboration of the protocol was conducted in four stages. 

In the first stage, a search in the literature was performed in order to access the main studies regarding LT for hCCA up to date. In the second stage, an outline of the protocol was designed by the first two authors and the last author, based on the study conducted at the Mayo Clinic^4^ ^,^ ^8^ ^,^ ^16^. In the third stage, 10 experts elaborated the last version of the protocol, adapted to the Brazilian reality. The last stage consisted of the protocol submission for approval in the National Transplant System (SNT - Sistema Nacional de Transplantes) of the Brazilian Ministry of Health.

Brazilian centers will be selected for inclusion in the multicentric research project and a total of 30 patients will undergo transplantation according to the criteria of this protocol, and will be referred to these centers by the SNT. Preoperative, intraoperative and postoperative data will be prospectively recorded on the REDCap platform^7^. The following pre-transplantation data will be analysed: age, gender, diagnosis of hCCA, staging examinations, tumor size, neoadjuvant chemotherapy and radiotherapy, diagnostic laparoscopy or laparotomy, anatomopathological analysis of the lymph node chains assessed in the staging surgery, time between diagnosis of hCCA and LT, and type of LT (deceased-donor or living-donor). The number of patients referred for LT evaluation, as well as the number of patients who effectively met the criteria and were included for undergoing LT and those who were excluded before the LT (due to not meeting the criteria or to contraindication after undergoing laparoscopy/laparotomy) will be assessed as well. After the LT, the following data will be analyzed: disease-free survival and overall survival in 1, 3 and 5 years; immunosuppression protocol; rejection episodes; and need for retransplantation. 

## RESULTS

**FIGURE 1:**
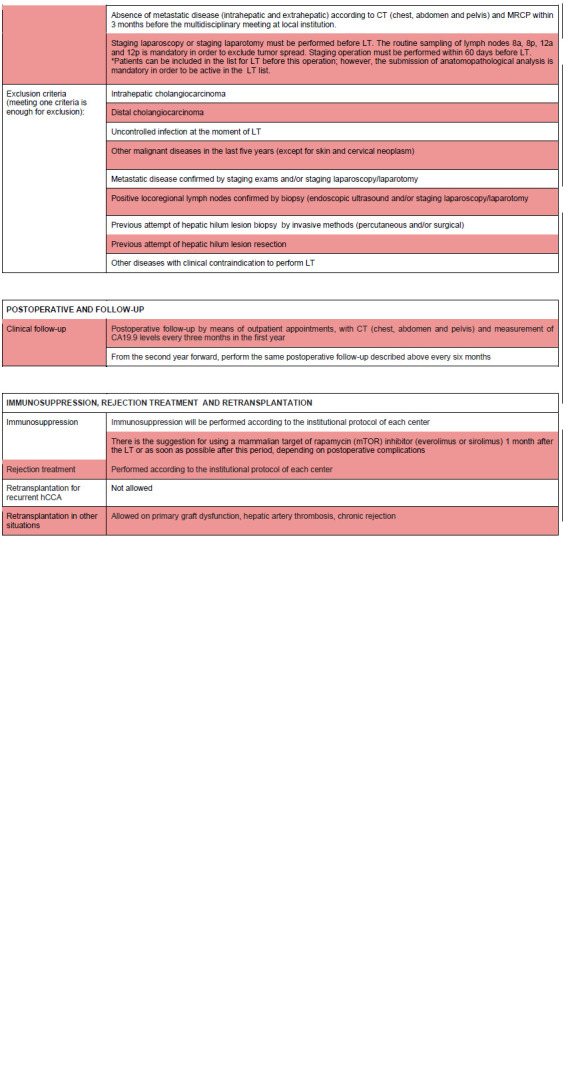
Protocol created by the authors for liver transplantation in hilar cholangiocarcinoma.

**FIGURE 2:**
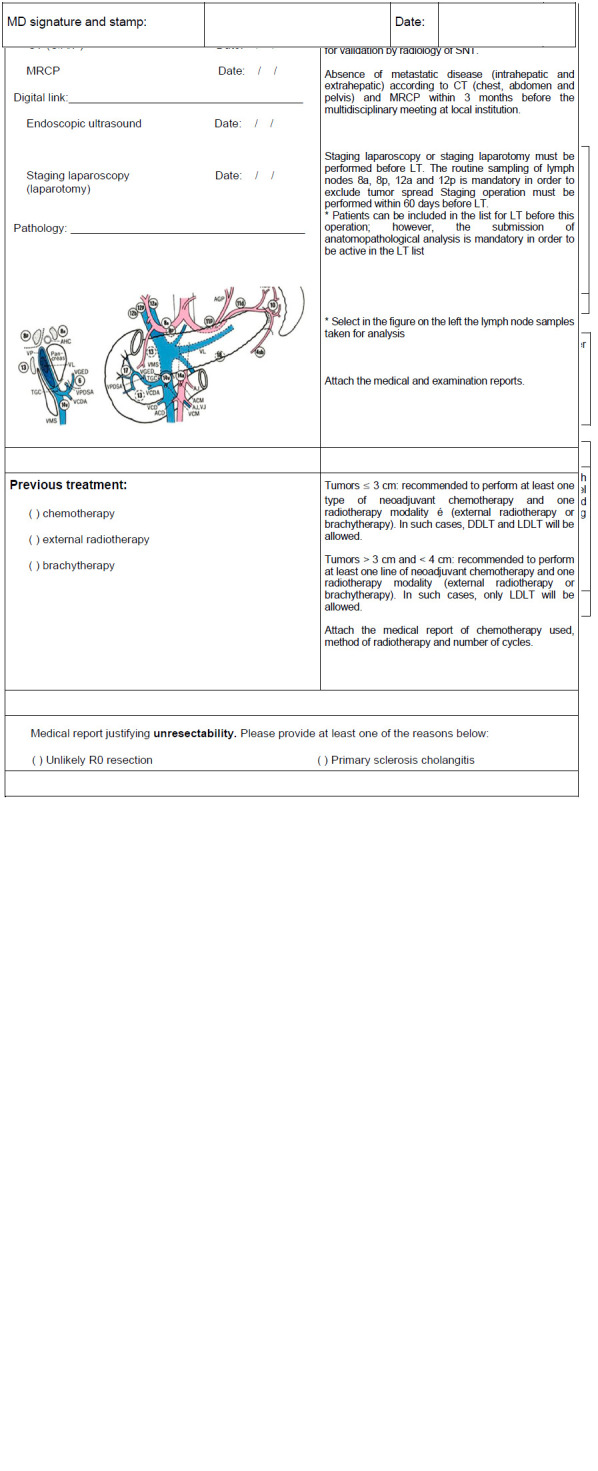
Document of SNT to be filled in to request a special situation for hCCA.

Figure 1 shows the LT protocol for hCCA proposed in this study by the authors (Figure 1). Figure 2 shows the document of SNT to be filled in to request a special situation for hCCA (Figure 2).

LT=liver transplantation; DDLT=deceased-donor liver transplantation; LDLT=living-donor liver transplantation; hCCA=hilar cholangiocarcinoma; SNT=national system of transplants; CT= computed tomography; MRI= magnetic resonance imaging; MRCP= magnetic resonance cholangiopancreatography

LT=liver transplantation; DDLT=deceased-donor liver transplantation; LDLT=living-donor liver transplantation; hCCA=hilar cholangiocarcinoma; SNT=national system of transplants; CT= computed tomography; MRI=magnetic resonance imaging; MRCP=magnetic resonance cholangiopancreatography

## DISCUSSION

In 1987 at the University of Nebraska, Sudan et al.^19^ introduced the concept of neoadjuvant therapy with improvement in long term results. In 1993, the Mayo Clinic group initiated a pilot protocol of neoadjuvant therapy for patients with unresectable hCCA or hCCA with primary sclerosing cholangitis (PSC). This protocol consisted of external radiotherapy (4500 cGy in 30 sessions) associated with a 5-fluorouracil (5-FU) bolus infection in the first three days of irradiation. Brachytherapy with Iridium-192 (2000-3000 cGy) was started 2-3 weeks after the end of external radiotherapy. Finally, patients were maintained on a 5-FU infusion pump or capecitabine orally until transplantation. All patients were submitted to a staging laparotomy before LT. Preliminary results of 11 patients were published in 2000 and were promising^4^. Results of this cohort (n=28) were updated in 2004 and showed an overall survival of 82% in 5 years^8^. The final result of this Mayo Clinic protocol study was published in 2005: 38 patients with an overall survival of 92% in 1 year, 82% in 3 years and 82% in 5 years^16^.

Since the creation of the Mayo Clinic protocol, other centers have published their experience using the same or a similar protocol. In 2012, a multicenter study carried out in the USA (12 centers) published a cohort with 287 patients - in tumors smaller than 3cm and with neoadjuvant therapy, in agreement to the protocol, disease-free survival of 69% in 5 years^3^ was achieved. In addition, an european multicenter study (21 centers) presented the results of LT in 159 patients selected according to the criteria of the Mayo Clinic, however without performing neoadjuvant therapy, having an overall survival of 59% in 5 years^13^. 

Tan et al.^20^ reviewed the current literature on the indication of LT for hCCA and emphasized that the waiting time before LT can be beneficial in selecting patients, consequently presenting superior results. In comparison to other LT indications, there is an increased risk of developing late artery and portal vein complications, probably due to irradiation. Neoadjuvant therapy combined with LT can achieve results comparable to resection in patients with unresectable hCCA at early stages and is the treatment of choice for patients with hCCA with primary sclerosing cholangitis. 

It is important to assess locoregional lymph nodes in order to avoid LT in cases with positive lymph node disease^11^ ^,^ ^15^. Just as in the protocols performed at the Mayo Clinic^16^ ^,^ ^20^, there is the suggestion of performing laparoscopy or laparotomy with evaluation of the lymph node chains mentioned above (8a, 8p, 12a and 12p). Finally, it is important to highlight the difficulty in determining the ideal time to perform this procedure, since the waiting list to receive an organ is very variable across the country.

The regulation of this protocol is now in progress in the SNT for validation in the Brazilian national territory^2^. 

## CONCLUSION

A liver transplantation protocol for hilar cholangiocarcinoma was created in order to standardize the treatment, as well as enable a better assessment of surgical results, disease-free survival and overall survival of these patients.
